# A Unique Method for Total Nasal Defect Reconstruction - Prefabricated Innervated Osteocutaneous Radial Forearm Free Flap

**DOI:** 10.1080/23320885.2018.1549494

**Published:** 2019-07-30

**Authors:** Uros Ahcan, Vojko Didanovic, Ales Porcnik

**Affiliations:** a Department of Plastic Surgery and Burns, Ljubljana University Medical Centre, Ljubljana, Slovenia;; b Department of Maxillofacial and Oral Surgery, Ljubljana University Medical Centre, Ljubljana, Slovenia

## Abstract

A 52-Year-old woman underwent a two-stage total nose reconstruction for complete nasal defect due to skin cancer. In the 1^st^ stage, innervated osteocutaneous radial forearm flap (“Neo nose”) was raised with the help of a 3D template. In the 2^nd^ stage, well vascularised “Neo-nose” was transferred to the face and covered with pre-expanded forehead flap.

## Background

Reconstructing functional and aesthetically pleasing nose has remained a challenging task since ancient times [1]. Regardless of the etiology (e.g. trauma, infection, tumours), nasal defect can be restored with a variety of autologous techniques (local flaps, cartilaginous or bony grafts, composite grafts, free flaps) or with the prosthetic implants [2]. Total or subtotal rhinectomy, resulting with severe facial disfigurement, requires complex reconstruction of three distinct anatomical layers: inner nasal lining, supporting midlayer framework and external skin. Nose inner lining remains the most difficult to reconstruct, with most of the reconstructive surgeons favouring microvascular free flap transfer [3–8]. For total nasal defects, reconstruction with osteocutaneous radial free flap alone or with combination with other local flaps, have been proposed [4, 9, 10].

One of the fundamental factors influencing the end result is the transfer of a free flap with well-vascularised inner and supporting midlayer framework at the same time. In achieving the desired shape and harmonious proportions, prefabrication of tissues can be shaped with 3D –laser scanning and the help of nose replica mold and custom made titanium coated cage. Furthermore, we think that innervation of the inner lining would improve patient’s quality of life even more, such as improving various nasal symptom perception and detection of hazardous external temperatures. The aim of this report is to present a novel approach of two-stage total nasal reconstruction with innervated prefabricated osteocutaneous radial forearm flap.

## Case report

We present a case of a 52-year-old woman, who was referred to the Department of Plastic and Reconstructive Surgery, University Medical Centre Ljubljana. She had undergone a total rhinectomy 4 years ago due to invasive squamous cell carcinoma. Nose pyramids, upper third of the upper lip, anterior part of the maxilla were removed. Patient had also selective neck lymph node dissection and needed no irradiation therapy. On presentation she wore a nose epithesis, which caused her psychological distress due to loose attachments, patient was depressed. She complained of severe pain inside nose mucous membrane especially in the winter months and had frequent chronic inflammations. We preformed innovated staged nose reconstruction.

**Figure 1.**
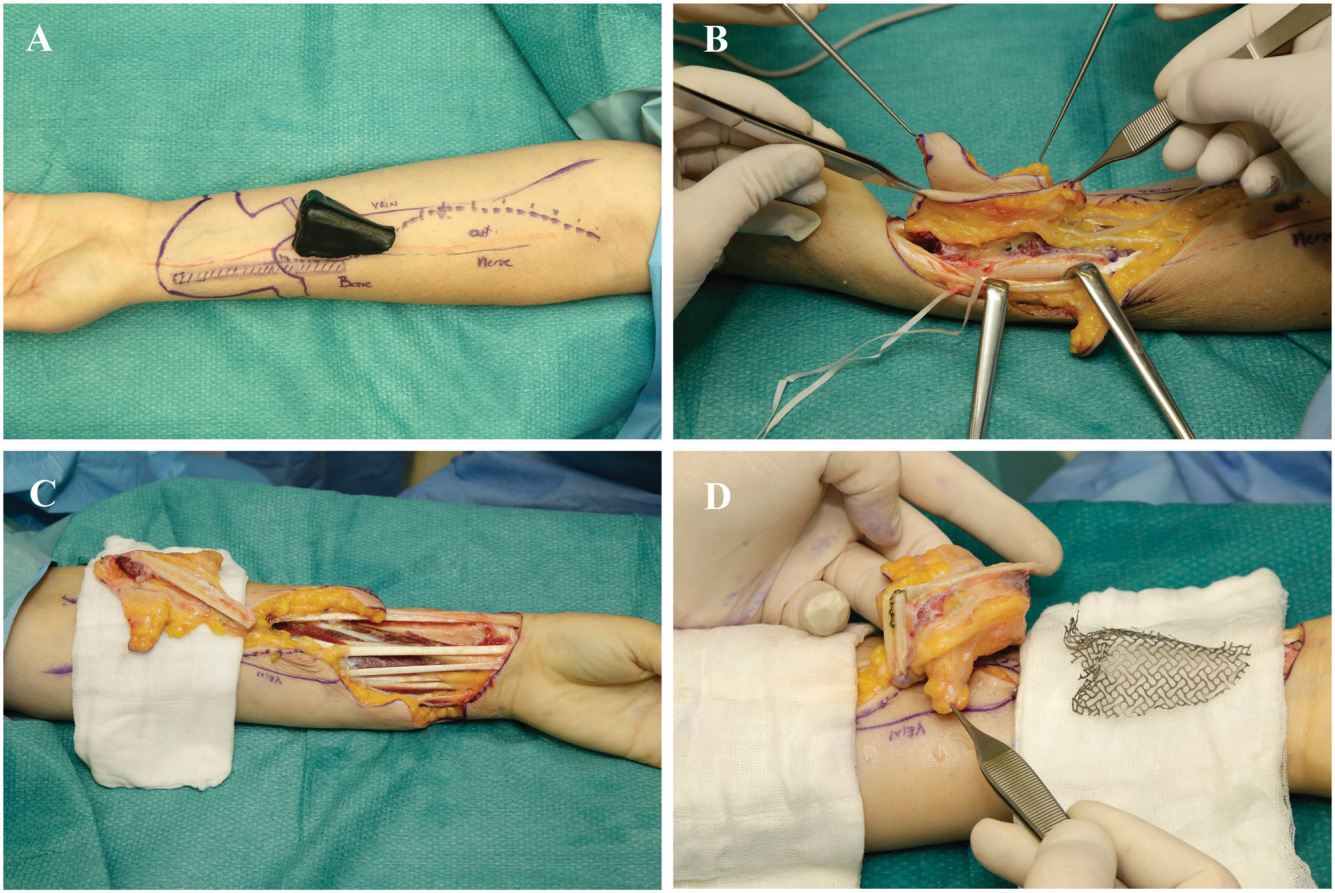
Intraoperative pictures from the 1st stage. Exact preoperative drawings on the patient forearm based on the rubber 3D nose mould (A). Flap was raised, osseous perforators to the radial bone were preserved (B). Osteo-fascio-cutaneous flap was raised (C) and radial bone was osteotomised on the cortical site and bone framework constructed using micro plates and screws (C). Please note that titanium material was hidden inside the bony groove.

**Figure 2.**
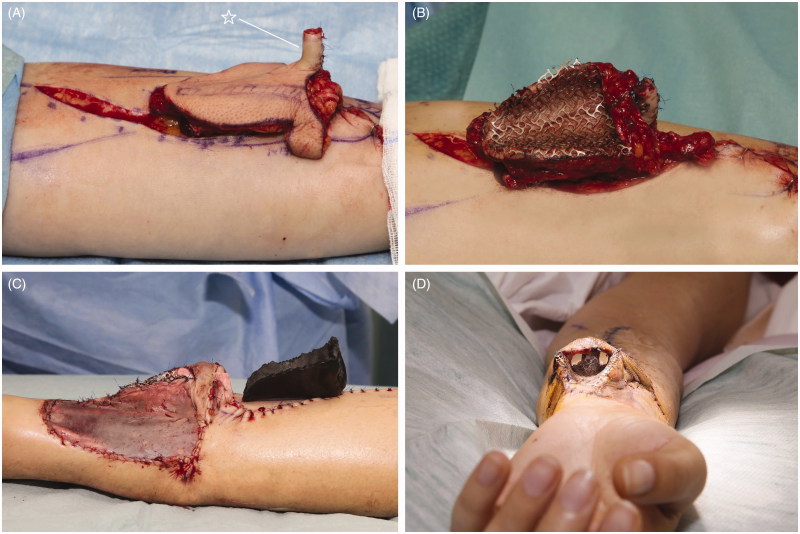
Intraoperative pictures from the 1st stage. Fascia and soft tissue were sewed around bone framework (A), and prefabricated titanium coated cage was used to line soft tissue inner lining (B). “Neo-nose” at the end of the 1st stage (C,D). * - columella

**Figure 3.**
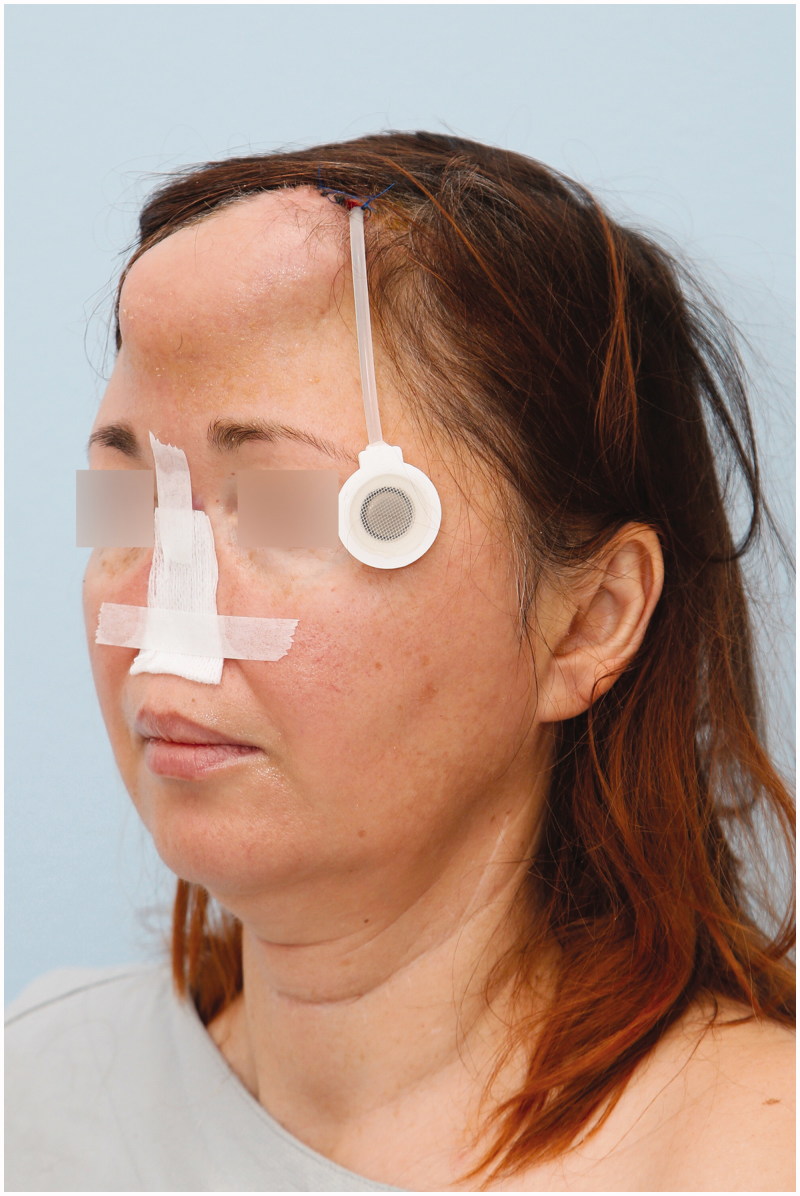
Patient with tissue expander before the 2nd stage.

**Figure 4.**
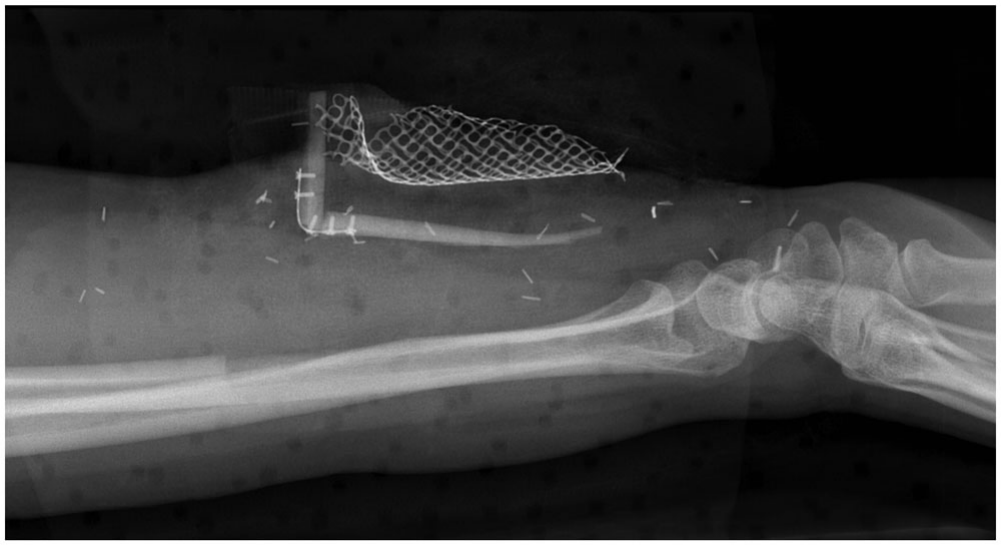
X-ray of the patient forearm after the 1st stage. Note the “L-shaped” bone framework and the position of titanium coated cage.

In the 1^st^ stage, an innervated osteocutaneous radial forearm free flap was designed based on a sterilised rubber 3D nose mold, which served as a template for skin incisions (Figure 1A). With meticulous dissecti on we preserved the osseous perforators to the radial bone, ensuring its vascular supply (Figure 1B, C). After radial osteotomy, an “L” shaped bone framework was reconstructed, using titanium micro plate and screws according to the preoperative measurements and 3D print (Figure 1D). Refined bone shaping enabled shaping of the columella and vertical bone thin. Well-vascularised bony framework was additionally covered with antebrachial fascia, further increasing vascularity. Prefabricated titanium coated cage was used to form soft tissues from the newly raised flap (“neo-nose”) (Figure 2A, B). Tissues were held in a rigid and anatomically correct position - representing nose inner lining. Titanium coated cage gave a desired support to the nasal vestibulum, tip, alae and the columella. A lateral antebrachial cutaneous nerve (*LABCN*) was dissected and preserved. When the “neo nose” was completed, it was reconnected back to the forearm (Figur*e* 2C, D). In this stage, a tissue expander was placed in the subgaleal plane on the forehead, which was later regularly filled with saline solution until its maximum. Patient was discharged without any substantial facial disfigurement, except of placed tissue expander on the forehead (Figure 3). She wore temporary nasal prosthesis until next stage. Figure 4 shows x-ray of newly reconstructed “neo nose” with its bony framework and titanum coated cage.

**Figure 5.**
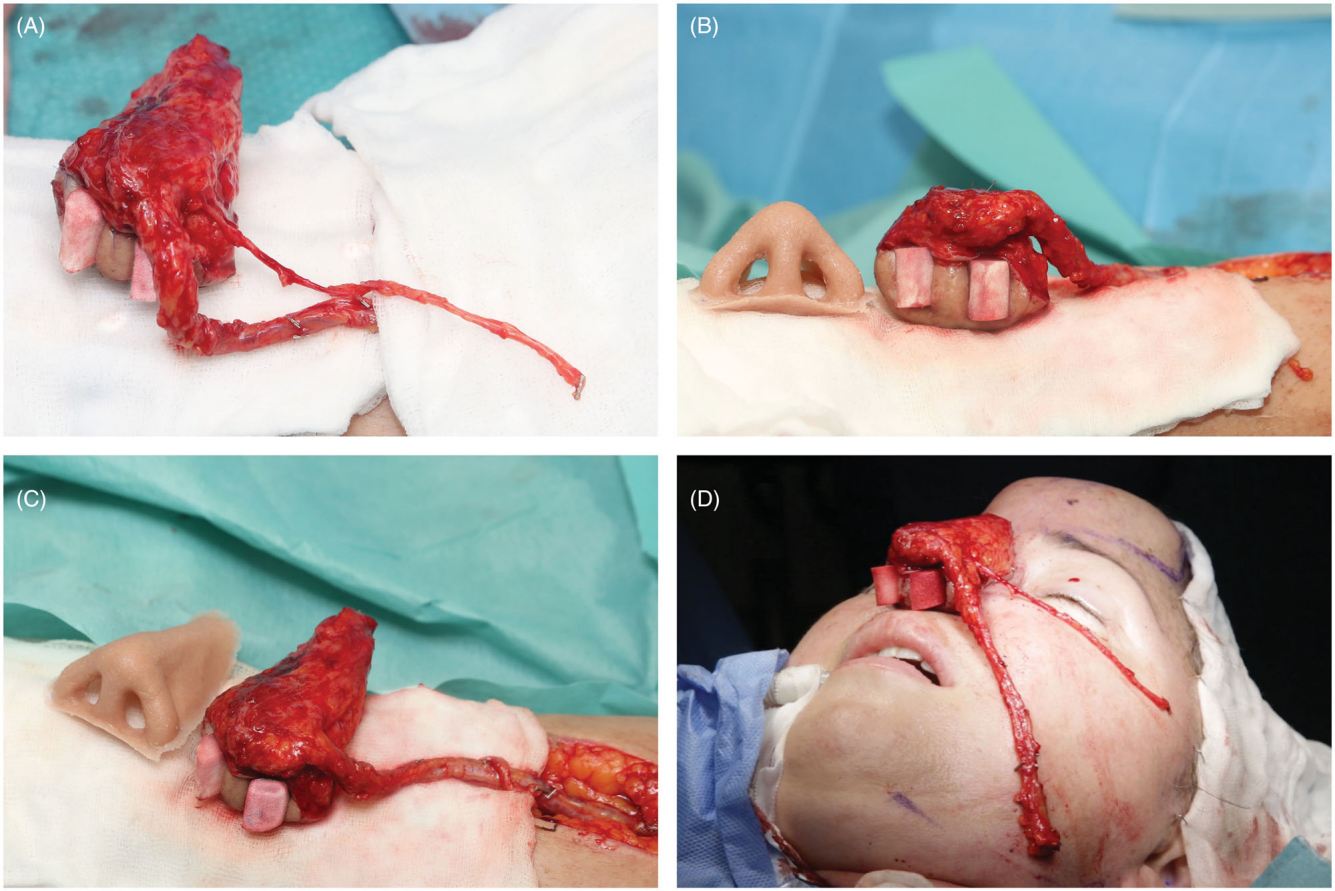
Intraoperative pictures from the 2nd stage. Innervated radial osteo-fascio-cutaneous flap (“Neo-nose”) was raised with a 12 cm long pedicle (A). Please note the LABCN included in the flap. “Neo-nose” in comparison to patient nose prosthesis showing nose inner lining harmonious proportion and likeliness to the epithesis (B, C). “Neo-nose” transferred to the mid-face. Donor vessels were anatomised to the facial vessels, donor nerve to the branch of the infraorbital nerve (D).

**Figure 6.**
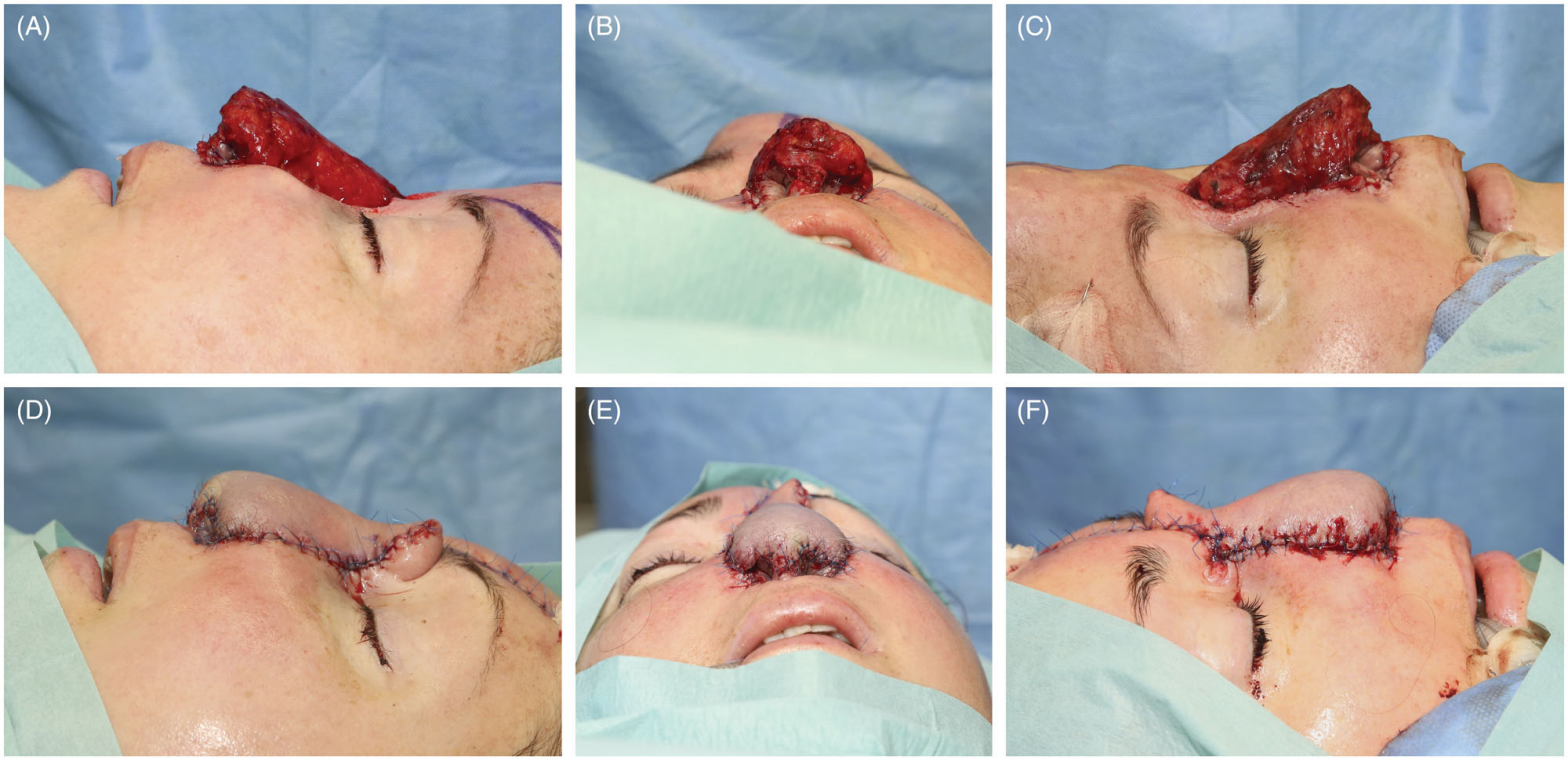
Intraoperative pictures from the 2nd stage. Patient with the “Neo-nose” before (A, B, C) and after final cover with the preexpanded paramedian forehead flap (D, E, F)

Five weeks after uneventful postoperative recovery, a 2^nd^ stage was performed. “Neo-nose” was re-raised with a 12 cm long vascular pedicle including a *LABCN* (Figure 5A). All shaped tissues (nose inner lining and supporting midlayer framework) were viable and with excellent vascular supply. After titanium coated cage was removed, tissues preserved their desired shape and remained appropriately firm (Figure 5B, C). Simultaneously, facial artery, vein and a nasal branch of infraorbital nerve were prepared. After tunnelling pedicle under the cheek, end-to-end anastomoses were performed using interrupted 8–0 sutures for the artery and nerve and running 9–0 sutures for the vein. Bone framework was fixed to the frontal bone and maxilla using titanium micro-plate and screws. In the same stage, tissue expander was removed, pre-expanded paramedical forehead flap harvested, rotated downwards and sutured over the “neo-nose” with interrupted sutures (Figure 6A–F). Small defects on the forehead and forearm were skin grafted. Patient was discharged home as all flaps were well perfused.

**Figure 7.**
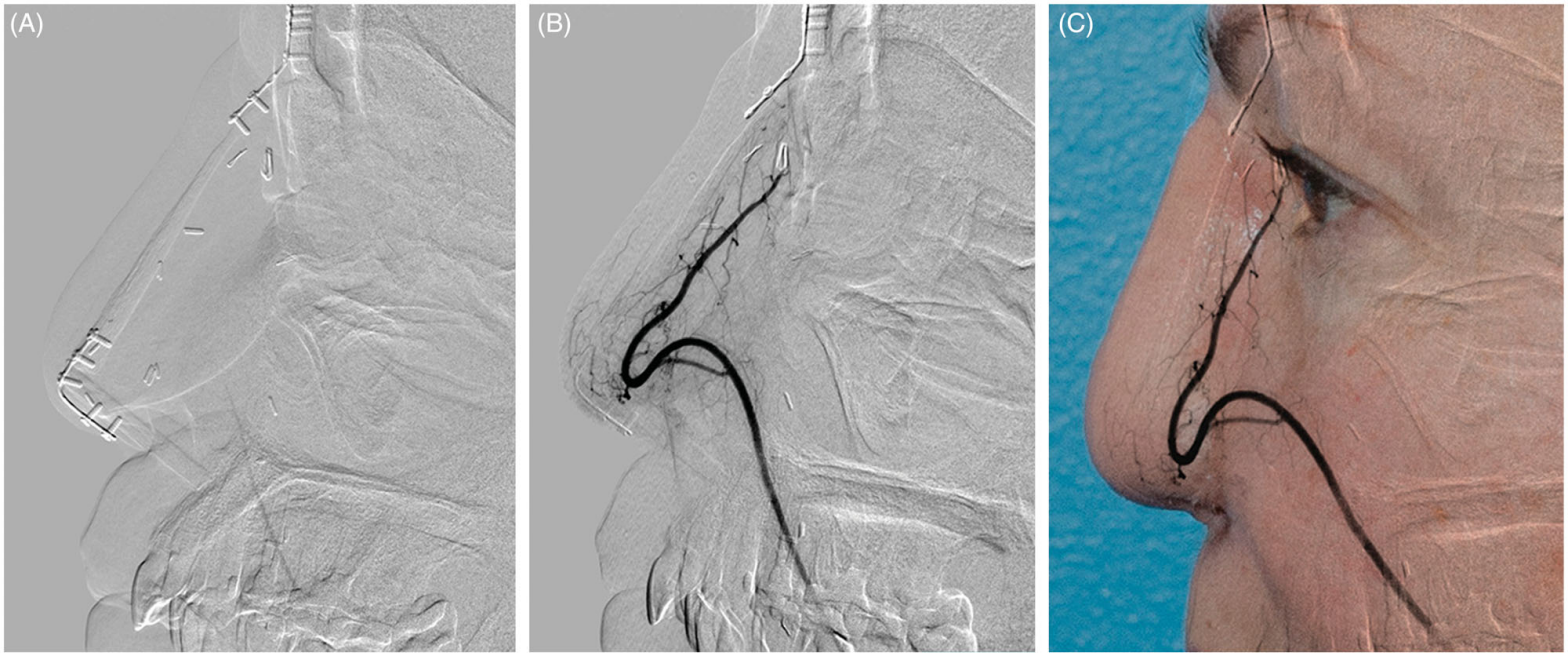
CT angiography of “neo-nose” vascularization. On the left side, picture shows nose just moment before contrast was injected into the pedicle (A, B). On the right side, CT angiography shows transparency to the actual patient face (C).

A CT scan of the “neo-nose” was performed 2 months after the surgery and showed signs of excellent vascularisation of “L-shaped” bony framework with no signs of resorption. CT angiography showed fully patent and prominent radial artery (Figure 7).

**Figure 8.**
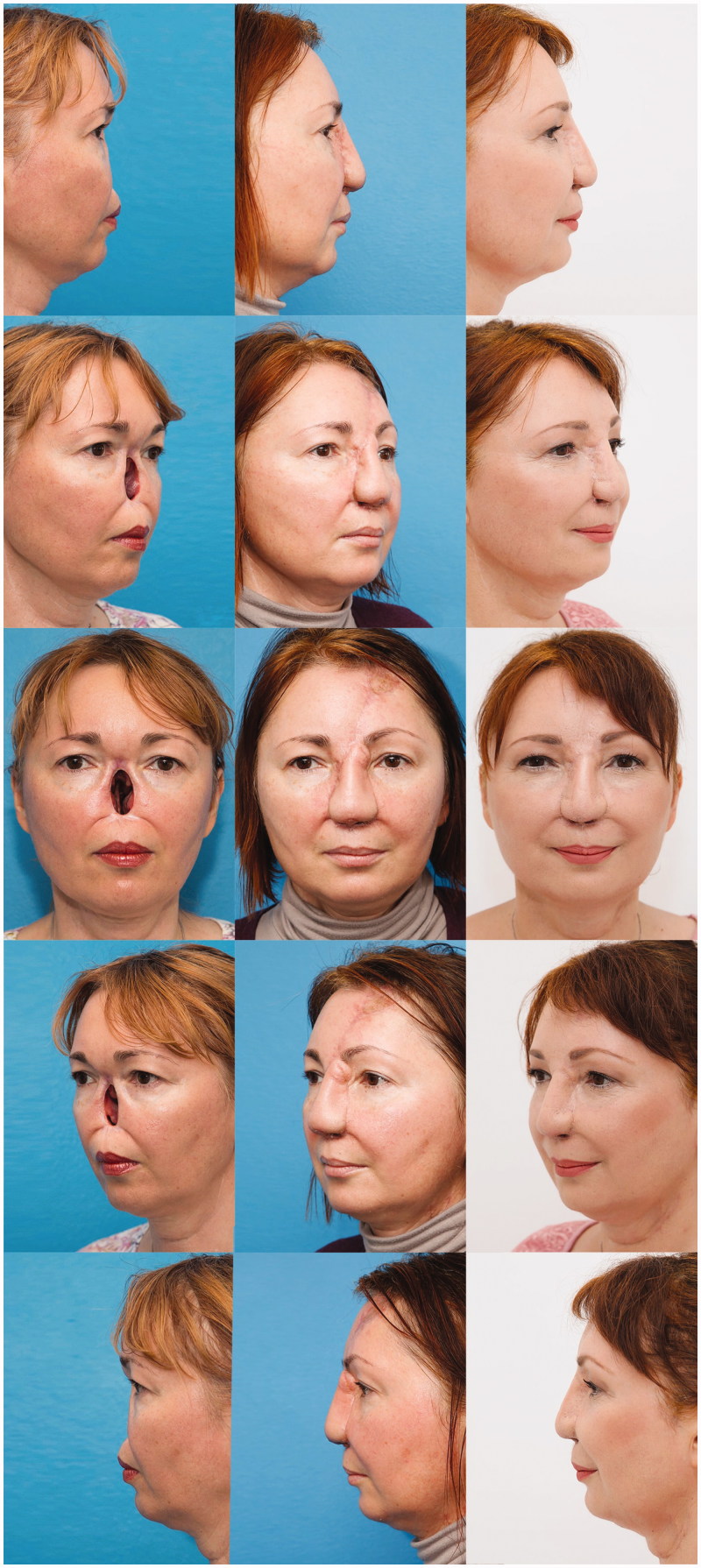
Patient before total nasal reconstruction (left column), after 2nd stage (middle column) and after additional minor corrections – final result (right column), using prefabricated innervated osteocutaneous radial forearm free flap and paramedian forehead flap.

**Figure 9.**
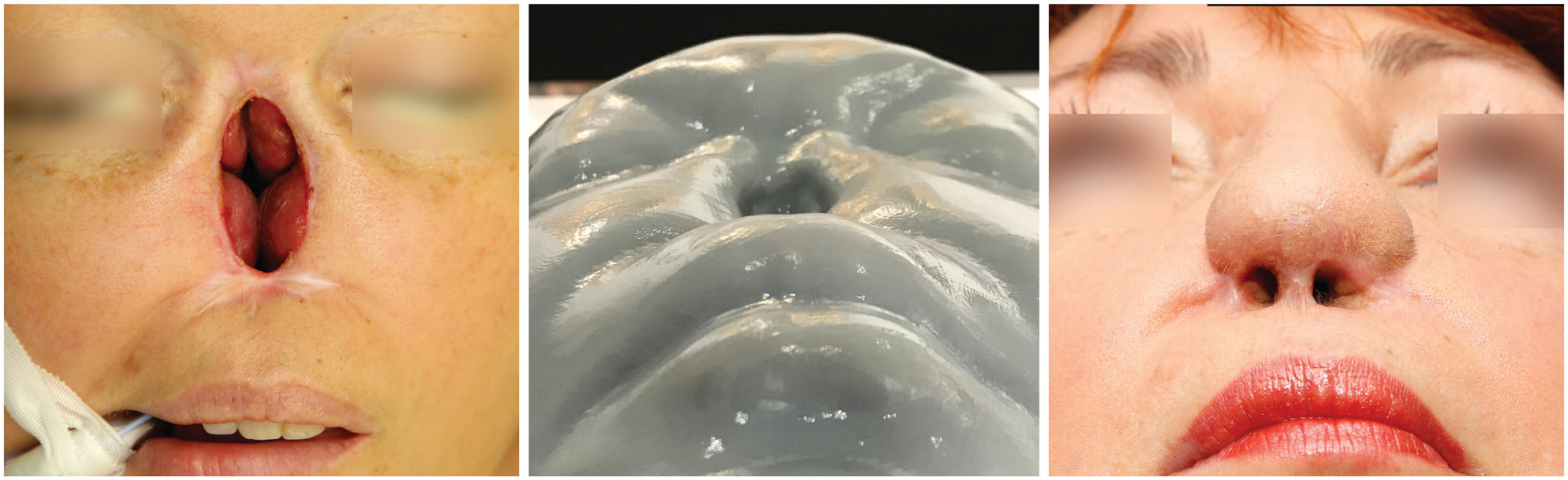
Bird’s eye view of patient immediately before 2nd stage. Worm’s eye view of patient’s 3D model of nose defect (middle picture) and patient final result (right picture).

**Figure 10.**
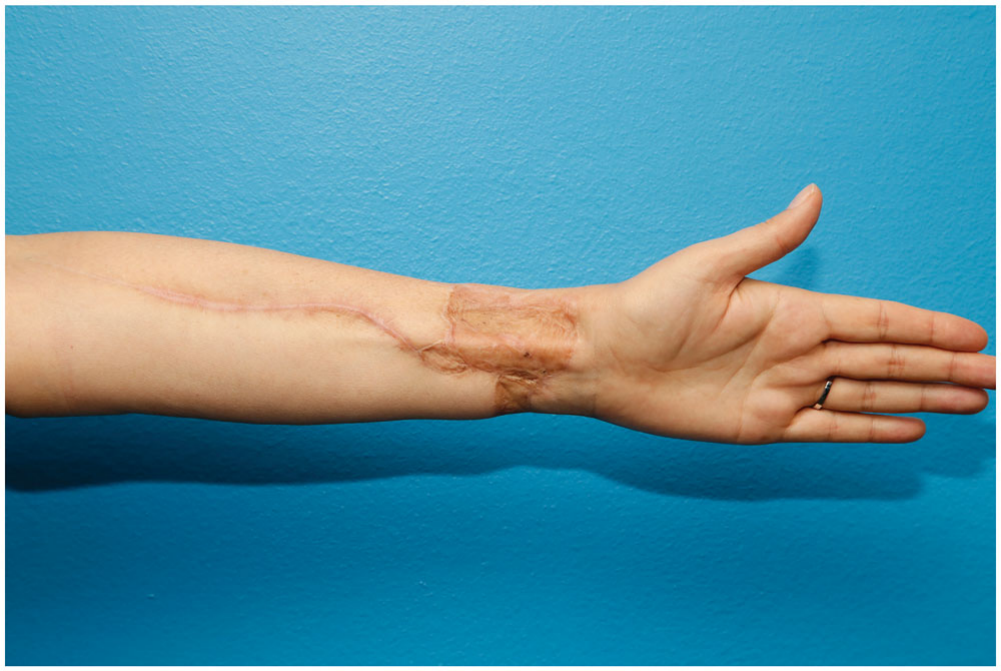
Patient donor site, 9 months after 2nd stage.

After only two surgeries, an aesthetically pleasing nose was reconstructed, which is a good basis for further minor corrections (Figures 8,9). Patient was extremely satisfied with a fully functional and aesthetical result, with minimal donor site morbidity (Figure 10).

## Discussion

Total or subtotal full-thickness nasal defects represent a considerable challenge for the reconstructive surgeon. It is now widely accepted that such defects preclude the use of local flaps and usually need composite microvascular free flaps for inner lining, appropriate suspension framework, accompanied by paramedian forehead flap for external coverage [3]. For inner lining – which is the most difficult to reconstruct - a variety of different free flaps have been described [6, 7]; among them, radial forearm flap is most widely used, yielding a reliable end result [3, 8, 11, 12]. Reconstruction using osteocutaneous radial forearm free flap, has been previously proposed by some authors [4, 9, 10, 13]. We believe it is a good reconstructive option and should be proposed to the patient with total nasal defect.

Traditionally, supporting nasal framework is created using non-vascularised bony or cartilaginous grafts [14]. In comparison to vascularised bony grafts, they do not have its own blood supply and survival depends only on surrounding tissue vascularisation. It is stated, that vascularised bone transfer has significant advantages over conventional bone grafting, due to preserved cellular viability [15]. Reconstructed vascularised bone has faster union rate and it tends to have lower absorption rate over time, giving long-term stability [6, 10]. It is now well-known that periostium is of vital importance for bone healing, and reduces incidence of non-union [16]. Another possible advantage of good vascularised flap reconstruction of a part exposed to external temperatures changes could be improved susceptibility to cold intolerance and discoloration, but further studies need to be performed to confirm this hypothesis. In our case, osteotomy was performed on the site of cortical bone, leaving vascularised periosteum intact, which obtained blood supply from the preserved osseous perforators. Another disadvantage of bony/cartilaginous grafts are additional donor site morbidity, whenever harvested. Long term complications and donor site morbidity for rib graft harvesting include warping and hypertrophic chest scarring [17].

Among all osteocutaneous flaps, radius is an excellent option for nasal supporting framework reconstruction because it is a thin bone with a strong corticalis easily susceptible to accurate cutting and fine shaping. Thin, viable bone is necessary to give support to future columella. After radial osteotomy, titanium micro plate and screws can be placed inside shallow bony groove on the cortical side, making them less prominent and minimising likelihood of future implant exposure. Thin radial bone was cut without leaving sharp edges, thus minimising radial bone fracture – an important donor site morbidity that needs to be considered.

Our method emphasises the importance of preoperative planning using a 3D laser technology. With the help of an exact replica of patient nose epithesis, skin incisions were made according to the markings, thus minimizing unnecessary tissue harvesting. A custom made titanium cage was used to enable reconstruction of nose inner lining with pre-defined harmonious proportions. Prefabrication of radial forearm flap was also reported by Sinha and colleagues, but they used non-vascularised grafts and prefabrication was not custom made [14]. In their case, an inner lining could be reconstructed with remaining nasal tissues and a radial forearm flap represented an external coverage - which was not optimal for skin colour and texture [14].

Titanium coated cage served as a template for nose inner lining. Skin and fascia were sutured around the cage and retained its shape when it was removed immediately before the 2^nd^ stage. To further improve nasal shape, cartilage grafts could have also been used for additional alar rim support. In the future, we would also consider this modification to our technique.

In our dissection we also incorporated a sensory nerve, allowing the possibility for inner layer nose innervations. A sensate osteo-fascio-cutaneous prefabricated free flap for inner lining nose reconstruction, to our knowledge, has never been described before. Normally, nose inner lining is innervated, which is a result of human evolution. In our opinion, sensate nose inner lining gives nose an important function and increases quality of life. Advantages are perception of potential dangerous external temperatures, faster perception of various symptoms related to upper tract infections or allergies and last but not least, faster detection of possible malignancy recurrence.

One of the mayor advantages of our unique method is favourable location of the “neo-nose” reconstruction. Forearm gives us sufficient working space with abundance of healthy tissues to use for reconstruction. After the 1^st^ stage, patient could normally wear nasal prosthesis leaving no facial disfigurement. Menick, a world known expert on nose reconstruction, proposed a five stage nose repair using a folded radial forearm flap, completed over the period of eight months. In a series of 12 patients, good to excellent results was obtained in 38% of patients [3]. Between the first two stages, which are two months apart, patient functional and aesthetic appearance was transitionally distorted, which probably represented great distress and decreased quality of life. In our case, the 1^st^ and main stage was performed on the forearm, and the patient was discharged without any substantial facial disfigurement, only leaving with a tissue expander on the forehead. She could normally use a nasal prosthesis until the 2^nd^ stage. Our method of nose reconstruction represented to the patient considerable less time of facial disfigurement thus increasing quality of life during reconstruciotn.

For external nasal reconstruction, we used a well-established technique with pre-expanded paramedian forehead flap [18]. Skin expansion increases flap surface, decreases skin thickness, increases tissue viability and gives less donor site morbidity. In our opinion, paramedian forehead flap is also one of the best options for external surface coverage because of its matching skin colour and texture. During 2^nd^ stage, we noticed excellent neo-nose vascularisation with full coverage of osteosynthetic material with viable tissue. We considered an option in which a full-thickness skin graft from pre-expanded forehead skin would be used as an external coverage, creating an already perfect end result with matching skin colour and texture. Potential advantages would include minimizing bulkiness that would otherwise be transferred with paramedian forehead flap. Because this was our first case in the series, we choose traditional and safe variant, using a flap instead.

Composite tissue allotransplantation (CTA) is another option for reconstructing total nasal defects. So far, face transplantation, among other types of CTA, had promising results, but with main disadvantage of taking harmful immunosuppressive drugs. We believe CTA is contraindicated in patients with a history of malignancy, as immunosuppressive drugs increases risk of neoplasms [19].

Our technique is reliable and precise and has yielded an aesthetically pleasing nose after only two surgeries. Further minor corrections include forehead flap division with glabella reshaping, flap thinning, alar groove defining, etc. The position of flap pedicle (“neo-nose”) is of vital importance as it could limits surgeon’s ability to freely reshape all aesthetic units of the nose.

## Conclusion

With this case report, we present a novel approach of total nasal defect reconstruction, using prefabricated innervated osteocutaneous radial free flap combined with pre-expanded paramedian forehead flap. Such reconstruction gives resemblance to nose prosthesis due to flap 3D prefabrication and long term stability due to well-vascularised osteocutaneous flap transfer. We hope this technique of nose reconstruction will become generally accepted option among reconstructive surgeons.
